# Staying true with the help of others: doxastic self-control through interpersonal commitment

**DOI:** 10.1080/13869795.2019.1641613

**Published:** 2019-07-15

**Authors:** Leo Charles Townsend

**Affiliations:** Department of Philosophy, University of Vienna, Vienna, Austria

**Keywords:** doxastic self-control, interpersonal commitment, avowal, collective belief, epistemic temptation

## Abstract

I explore the possibility and rationality of interpersonal mechanisms of doxastic self-control, that is, ways in which individuals can make use of other people in order to get themselves to stick to their beliefs. I look, in particular, at two ways in which people can make interpersonal epistemic commitments, and thereby willingly undertake accountability to others, in order to get themselves to maintain their beliefs in the face of anticipated “epistemic temptations”. The first way is through the avowal of belief, and the second is through the establishment of collective belief. I argue that both of these forms of interpersonal epistemic commitment can function as effective tools for doxastic self-control, and, moreover, that the control they facilitate should not be dismissed as irrational from an epistemic perspective.

## 1. Introduction

Amongst the epistemic virtues perhaps the most prized are openness and sensitivity to evidence. As believers, we should expose ourselves to relevant evidence, and form, revise or suspend our beliefs in response to what that evidence demands. We should not leap to judgment on the basis of scant evidence, or form beliefs that contradict the facts at our disposal, and we should not be stubborn when our evidence demands that we change our minds. The trouble, however, is that it is not always clear, from the first person perspective, just what our evidence is and what it demands: what seems to be evidence might turn out to be mere distortion, illusion and temptation. Our thinking is pervaded by cognitive biases, and unduly influenced by all manner of emotional, social and situational pressures – none of which are easily identified and neutralized by the agent herself as she makes up her mind or as she maintains her attitudes.

Considerations such as these suggest that, alongside openness and evidential sensitivity, there is also a place for something like *doxastic self-control* in our cognitive lives. Like practical self-control, doxastic self-control is needed to overcome certain forms of characteristic weakness. Such control might be exerted in the *formation* of belief, as when a person takes pains to gather all the relevant evidence before she makes up her mind – rather than judging on the basis of a small, perhaps convenient, portion of it. And it might be exerted in the *maintenance* of belief, as when a person takes steps to guard her rationally-held beliefs from “epistemic temptations” (Paul 2015a) or “distorters” (Pettit 2016b). It is this latter, diachronic form of doxastic self-control that will be my specific focus here.

My aim in this paper is to build on recent work by Sarah Paul, who has argued that there is a place for doxastic self-control in our cognitive lives.^1^ But whereas Paul sees such self-control as a fundamentally *individual* achievement, I wish to examine *social* tools of doxastic self-control – ways in which we might enlist the help of others in order to stick to our beliefs. More specifically, I explore whether people can stick to their beliefs by making *interpersonal epistemic commitments*, and whether doing this could count as an epistemically rational strategy for doxastic self-control.

The reason for this focus on interpersonal epistemic commitment is that interpersonal commitment is a familiar tool for getting oneself to stick to one’s plans and intentions, that is, for *practical self-control*. For example, there is evidence to suggest that one way to get yourself to do something is to *promise* somebody that you will do it; another is to make a joint plan or *agreement* to do it with others.^2^ Promises and agreements are two forms of interpersonal commitment, two ways in which people make themselves responsible or accountable to others, and it is this that appears to make them apt tools for practical self-control. In making a promise or an agreement one gives oneself added incentive to stick to one’s intention, because other people are now authorized to hold one accountable should one fail to follow through.

Since promises and agreements appear to be effective tools for practical self-control, we may ask whether there are analogous tools available for doxastic self-control. Here I explore two such candidates, which can be seen as the counterparts of promising and agreements: belief-avowals, and the establishment of collective belief. Drawing on work from Philip Pettit on avowal, and Margaret Gilbert on collective belief, I will suggest that both these ways of fortifying one’s beliefs might, in principle, be effective tools of doxastic self-control. To end off, I consider the further question of whether they can also be considered rational mechanisms of doxastic control. I suggest that although neither of these mechanisms would be deemed rational by the lights of a strict evidentialist, they can nonetheless be considered rational from a slightly more relaxed perspective, since they do seem capable of promoting true, responsible believing.

## 2. Doxastic self-control

### 2.1. Doxastic weakness

To get a sense of what is meant by doxastic self-control, it may be helpful to consider its negative counterpart, what could be called *doxastic flimsiness*. Someone is flimsy in belief when she allows non-evidential factors to unseat her rationally-held beliefs. To illustrate this, consider the following two examples.

SUSCEPTIBLE GAMBLER: Suzie is a bright, diligent student. In her critical reasoning class she learns of the “gambler’s fallacy”, the false belief that, for example, the prospect of a fair coin toss coming up heads becomes more likely after a series of tails. She becomes fully convinced that the gambler’s fallacy is indeed a fallacy, and is even able to demonstrate this by applying Bayes’ theorem. However, when Suzie decides to visit the local casino with some friends, she finds that this conviction (that the gambler’s fallacy is a fallacy) begins to shrink from view. She does recall having been persuaded of it in her critical reasoning class, but now amongst the bright lights and the loud music of the casino she cannot recall the grounds for that conviction. In fact, as she stands at the roulette table, the very opposite conviction takes over: it just seems obvious to her that the long string of reds makes black a good, almost sure bet. Later that evening, reflecting on her lost savings, Suzie curses herself for falling victim to the fallacy. Once again the fallacious character of the gambler’s fallacy is clear to her. “What was I thinking?!” she says to herself. (Adapted from McGeer and Pettit 2009).

UNDERCONFIDENT PHILOSOPHER: Suzie is now an early-career philosopher. She has developed a well-worked out view (p) on a niche topic, and is ready to present the view at a large philosophy conference. Suzie not only has a powerful argument for her view that p, but has considered many possible objections to p and has solid replies to all of them. In short, she wholeheartedly believes that p, on the best possible philosophical grounds. Nevertheless, when it comes time to give her presentation, Suzie notices a number of famous, and notoriously disagreeable, older philosophers in the audience. Though none of these philosophers even work in her field, she anticipates that they will take issue with her view that p and vehemently oppose her – just for the sport of it, as it were. And she is right: the question-and-answer session after her presentation sees these luminaries bombarding Suzie with crude, ill-informed objections. But in the moment Suzie fails to see these objections for what they are: in her nervousness and exhaustion, she gives these famous philosophers too much credit, and as a result, begins to backtrack on many of her key claims, and ultimately retreat into uncertainty with regard to p. Later, going over her notes from the session, Suzie sees that these famous philosophers’ objections do not threaten her view at all, or that she has ready replies to them. So she returns to her wholehearted belief that p, but now feels profound disappointment and regret that she was not able to stick to her guns when under fire. (Adapted from Paul 2015a, 655).

There are a few things worth highlighting in these examples. First, it is assumed that in both cases Suzie really does start out with the beliefs in question – that she really believes the gambler’s fallacy is a fallacy, and that she really believes her philosophical account, p. Perhaps some would want to deny this, thinking that Suzie’s subsequent behavior shows that she never had those beliefs in the first place. But I think this is an unreasonably onerous requirement on belief: there is nothing in the concept of belief itself that requires this level of robustness or constancy. People routinely change their minds, or have their minds changed, without this impugning the status of their erstwhile attitudes.

The second feature worth noting is that Suzie’s failure in both cases is her failure to maintain those original beliefs, rather than merely her failure to act in accordance with them. So it is not that Suzie, in the gambling example, simply acts against her currently held better judgment, or that she, in the philosophy conference example, merely voices concession to the famous philosophers’ objections in order to appease them. Rather, in both cases, *she undergoes a change of mind:* she actually starts to think that black is more likely after the run of reds, and that p is not well-supported after all. Of course, these changes of mind are only short-lived blips, since once she leaves the casino table, or the lectern, she quickly reverts to her original belief. Still, however fleeting they may be, it is crucial to recognize them as genuine changes of mind.

This second feature is especially worth highlighting because it helps to distinguish the phenomenon I am exploring from another phenomenon that has received some attention under the banner of “doxastic self-control” – namely, *epistemic akrasia*.^3^ To be akratic in the practical sense is to act against one’s better judgment; similarly, epistemic akrasia refers to believing against one’s own, concurrently held, better judgment. Whether we can be akratic in this epistemic sense is controversial: many philosophers are skeptical about the very possibility of epistemic akrasia. But I can happily set that controversy aside for present purposes. This is because the kind of doxastic weakness I am concerned with here is not a matter of believing against one’s concurrent judgment. On the contrary, in both cases, Suzie’s beliefs at every stage are in alignment with how she judges. The problem, then, is not the synchronic problem of getting one’s beliefs to conform to one’s judgments but rather the diachronic one of maintaining one’s belief (and one’s judgment) in the face of non-rational “disrupters”. When Suzie chides herself for falling victim to the gambler’s fallacy, or when she feels regret that she was unable to stick to her guns in the Q&A, it is precisely her failure to maintain belief that she laments. And it is this kind of failure that doxastic self-control is meant to remediate. To exercise doxastic self-control is to stick by your beliefs in the face of disruptive factors – to have, as Paul (2015a) puts it, “the courage of your convictions”.

### 2.2. The possibility of doxastic self-control

Even if you find these examples convincing, you may still be concerned about the proposed remedy for such doxastic flimsiness: doxastic self-control. To display doxastic self-control is to effortfully maintain belief in the face of “epistemic temptations.” But this talk of effort, control, and even temptation may seem ill-applied in the realm of belief. After all, it is widely agreed that we cannot voluntarily control our beliefs – that we cannot believe “at will”.^4^ So how could we possibly exercise doxastic *self-control*?

In response to this concern it may be helpful to clarify just what kind of control over belief is at issue. Following Alston (1988) we can distinguish three different ways in which a believer might be thought to be able to control or affect her beliefs: through *direct intentional control*, through *indirect intentional control*, and through *doxastic influence*.^5^ Direct intentional control over belief would be a matter of forming or maintaining a particular belief simply by deciding to – that is, by carrying out an intention to form or maintain that belief. Though some philosophers hold that we have this kind of control over our beliefs (at least in certain cases), this view not widely held^6^ – and it is this view that non-voluntarists typically have in mind when they argue that believing at will is impossible. In contrast to this, indirect (or “long-range”) intentional control over belief is a matter of controlling one’s future beliefs by means of some intermediate intentional activity. As numerous philosophers have pointed out, this form of control is one people can and do exercise.^7^ For instance, we can strategically induce certain beliefs in ourselves by various effective means (for example, by means of hypnosis, or by joining a cult), and, equally, we can guard against other, unwelcome beliefs arising (for example, by avoiding certain literature or people). Finally, the third form of doxastic control is “doxastic influence”: this is a matter of intentionally acting in a way that one knows will profoundly affect one’s beliefs, though one does not know which beliefs will be affected and how. Clearly, we have a great deal of such doxastic influence, since we can decide what things to inquire about, which data or evidence we take into account, which evidential standards we apply, and how fastidiously we apply those standards.^8^

In terms of this distinction, doxastic self-control should be classified as a special form of *indirect intentional control* over belief. Doxastic self-control is a matter of taking intentional steps in order to maintain one’s current belief that p, where this means finding ways to resist the urge, brought on by non-epistemic factors, such as emotional, social, or environmental pressures, to re-open inquiry into the question of whether or not p. It is to stick to one’s guns in the face of epistemic weather – to continue to treat as settled a question one has previously settled for oneself, despite the onset of unsettling factors. Although the question of whether to open or re-open inquiry into some topic is usually a matter of doxastic influence (since one does not know in advance how a given inquiry may affect one’s beliefs), the phenomenon under discussion is somewhat different, because it involves deliberately preventing oneself from re-opening inquiry in order to maintain a given belief. So it is not mere influence but rather a matter of *control* over belief. And it is clearly not a matter of *direct* intentional control over belief, since it is not a matter of coming to believe that p simply by deciding to. Hence the concerns that motivate doxastic non-voluntarism, which are typically directed at direct intentional control over belief, need not cast doubt on the possibility of doxastic self-control.

### 2.3. The rationality of doxastic self-control

Let us assume, then, that doxastic self-control is possible. Still, one might remain skeptical about whether it is ever conducive to epistemic rationality to get oneself to stick to one’s beliefs. Indeed, it might seem that what I am calling doxastic self-control actually refers to a form of irrationality or epistemic vice – doxastic dogmatism or stubbornness.

To appreciate this skeptical worry, consider more closely the notion of “epistemic temptations” or “distorters” that I have been using to stress the need for doxastic self-control. These are non-epistemic factors, such as emotional, social or situational pressures, that threaten to unsettle belief. But it is important to note that when these factors pose their threat, their status *as non-epistemic* is not clear to the agent in question. For of course if the agent already knew these factors were non-epistemic then they would pose no real threat to her extant beliefs, and hence there would be no need for her to exercise doxastic self-control. The agent could simply dismiss these factors as *mere* temptations or distortions.

What this means is that doxastic self-control is a matter of sticking to one’s beliefs in the face of factors that, though actually non-epistemic, nonetheless do seem to one, for a time, to undermine one’s beliefs or to unsettle them. But how could this be anything other than an abject failure to respond properly to (what one takes to be) evidence; in other words, how could this be anything other than doxastic dogmatism?

In response to this worry, Sarah Paul emphasizes that the kind of “sticking to belief” we are concerned with here is not a matter of maintaining one particular view over the course of an *ongoing, open* inquiry. Rather, it is a matter of resisting the urge to *re-open* inquiry into some matter that is relevant to one’s extant beliefs. So it is not quite right to characterize these “epistemic temptations” or “distorters” as (seeming) evidence pertaining to an open inquiry – which would mean that exercising doxastic self-control is always a matter of believing against (what one at that moment takes to be) the evidence. Instead, epistemic temptations are factors that affect us prior to questions of evidence, factors that shake our confidence, unsettle our beliefs, and thus leave the field open for new, perhaps unwarranted, beliefs to take their place.

What this suggests is that sticking to one’s belief is not, or not always, a matter of stubbornly clinging to belief in face of contradictory evidence. This is precisely what the examples above are meant to suggest, that doxastic self-control can promote rational, responsible believing – that is can be, as it were, a matter of *staying true*. But it is important not to overstate this point, and especially not to give the impression that sticking to belief by suppressing factors that threaten to shake one’s confidence is a generally rational or laudable strategy. For often this kind of sticking to belief, while not a matter of believing against the evidence, does nonetheless deserve to be called out as a form of stubbornness – a matter not of staying true but of simply *holding fast*. Sometimes the factors that prompt us to reconsider questions we have previously settled for ourselves should be heeded, rather than silenced. So while we should recognize that there is a place for doxastic self-control in our epistemic lives, it must also be acknowledged that there is a fine line between this virtue and the corresponding vice of doxastic dogmatism: those who *stick to their beliefs* should be wary of becoming too *stuck* in them.

So far I have followed Paul in suggesting that exercising doxastic self-control – finding ways to stick to one’s beliefs in the face of non-rational factors that threaten to unseat them – is both possible and sometimes rational (or at least not necessarily irrational). In other words, doxastic self-control can be a matter of *staying true*, rather than simply *holding fast*. But Paul does not devote much attention to the further question of *just how* people might succeed in exercising doxastic self-control – and especially how they might do so while simultaneously guarding against doxastic dogmatism. Her primary suggestion is to say that, when we are faced with epistemic temptations, we can opt to defer to an earlier judgment rather than judging anew, this being the prerogative of someone who sees herself as “occupying a genuinely diachronic first-personal perspective”, rather than only a “synchronic, present-directed perspective” (Paul 2015a, 657).

In what follows I will explore some alternative ways in which doxastic self-control might be exercised. More specifically, in contrast to the kind of *intra*personal mechanism discussed by Paul, I will spend the remainder of the paper exploring the prospects for *inter*personal mechanisms of doxastic self-control.

## 3. Pettit: fixing belief through avowal

In recent work, Philip Pettit has drawn attention to the ways in which our discursive relationships with other people can bring order and stability to our mental lives. Apart from those relationships our inner mental lives would be messy, tumultuous affairs, constantly subject to change and rife with contradictions and tensions. According to Pettit, it is our social needs – especially our need to rely on others, and have them rely on us – that help us to bring order to this “carnival of passing states” (Pettit 2016a, 21). This is because, in the course of shared human life we are called upon to represent ourselves to others, including representing ourselves as people who have certain attitudes – who want certain things, who hold certain things true, and who seek certain goals. When we respond to that call, and express our desires, beliefs and intentions to others, the very fact of doing so creates social pressure for us to maintain those attitudes.^9^ In this way, according to Pettit, our *inter*personal relationships allow us to achieve a level of *intra*personal stability:

You fasten on the attitudes you avow and pledge in determining who you are, letting them emerge as fixed points by which others can orientate in their dealings with you. The self you authorise others to assume—the bespoken, beholden self—has a robust and even constant character that stands in contrast to the shapeless, shifting nature of the undisciplined mental life (Pettit 2016a, 20).

At the heart of Pettit’s view is a distinction between two quite different ways of communicating one’s attitudes to others: by “reporting” them and by “avowing” them. To report a state of mind is simply to say of oneself that one is so minded – in much the same way that one might report the attitude of another person. But people do not typically report their attitudes to one another; instead, the standard way of communicating one’s attitudes is by means of avowal.^10^ This is what we do whenever we assert that p or assert that we believe that p. The crucial difference between avowing and reporting belief lies in the *commitment* the speaker makes. When you avow an attitude of belief you lay claim to a certain authority, you authorize others to assume that that is your view. This means that if you do not maintain the attitude then you will face serious reputational costs.

Pettit cashes out the commissive character of avowal with reference to the notion of “precommitment”:

Such acts [of avowing an attitude] naturally count, by ordinary criteria, as acts of commitment in which I put my good name on the line. But they need only be commitments in the strategic, game-theory sense of precommitments […] In making a precommitment, say to performing a certain action, I place a side-bet on doing what I say I will do, where I stand to lose my stake should I fail to do it. By analogy, in making an avowal […] I bet on myself to display the attitude avowed (Pettit 2016b, 226–7).

This way of characterizing the commitment involved in avowal is significant for the present discussion, because precommitments are well-known tools for practical self-control – they are ways of manipulating your own future options and incentives so as to lock yourself in to a certain course of action. The archetypal precommitment was Ulysses’s act of binding himself to the mast of his ship so that he could hear the Sirens’ song without the risk that he would succumb to the temptation to change course. But precommitments need not always involve depriving oneself temporarily of agency, in the Ulyssean fashion. They can work in subtler ways, by introducing penalties or social costs for failing to fulfill your intentions. For example, there are apps that will punish you when you procrastinate on your work computer, or fine you when you try to phone your ex after 2am. And a precommitment-based strategy may also involve recruiting other people, with or without their knowledge or consent, into some kind of policing role. This is why people who are prone to procrastination sometimes choose to work in the company of others, rather than in the judgment-free zone of a secluded office.

Pettit’s central claim, then, is that the avowal of belief functions in the same basic way, raising the stakes for the speaker, placing her more firmly on the hook, and so giving her a powerful reason for sticking to her belief in the face of any would-be epistemic temptations. The practice of avowal thus has a built-in steadying mechanism, one which is mobilized in producing the “bespoken, beholden self”.^11^ The beliefs we bespeak, by means of avowal, are those to which we are then beholden, since others are authorized to hold us accountable for them. If we are too flimsy as believers, if we avow a belief one moment, then groundlessly backtrack the next, we will suffer serious reputational harm – we will not succeed in proving ourselves reliable interlocutors.

Is it possible, then, to *harness* this built-in steadying mechanism as a part of a conscious strategy of doxastic self-control? Pettit himself is not explicit on this point, though there would appear to be nothing in his account that would preclude that possibility. In fact, he sometimes implies that using other people for the purposes of achieving mental stability is something we all do all the time:

we each make good use of the reputational pressures that others bring to bear on us when they form expectations, by our license, about what we will think and do. Those pressures force us to be careful to advertise only attitudes we can live up to and to be careful about living up to the attitudes we advertise (Pettit 2016b, 306).

In fact, it is not difficult to think of examples in which the reputational pressures associated with avowal appear to be purposefully exploited in order to get people to stick to their beliefs. In many such cases the use of these pressures is institutional. Think, for example, of religious rituals that involve new converts declaring their belief to a congregation, an act that surely makes it harder for them to waver in their faith later on. And there are less institutionalized cases too, such as when holders of unpopular opinions openly declare their stance, and so make it harder for themselves to abandon that stance later. In these cases, avowal functions as a kind of doxastic precommitment – a matter not so much of binding oneself to the mast as nailing one’s colours to it, steeling oneself against future disrupters of the belief by openly declaring it.

## 4. Gilbert: doxastic coercion through collective belief

Whereas Pettit’s account of avowal involves the idea that the speaker makes a commitment *towards* her audience, the next proposal I want to examine involves a different sort of interpersonal commitment – one made *along with* others. The proposal is Margaret Gilbert’s notion of “joint commitment”, and specifically the sort of joint commitment that Gilbert claims is involved in the phenomenon of “collective belief”.^12^

According to Gilbert, a collective belief is formed whenever two or more people express to one another their readiness to let some proposition p stand as their view, that is, the view of them as a group. It might happen, for example, by my saying “Wow, those spring rolls were amazing” and you responding, “Yes, they certainly were”. According to Gilbert, what is going here is *not* that I am letting you in on my view, that the spring rolls were amazing, and you are responding by letting me know that you of the same mind. Rather, I put out the proposition *the spring rolls were amazing* as something we might go in for together. So it is as though, in saying “Wow, those spring rolls were amazing” I extend to you an invitation to join with me in this enterprise: of our believing the spring rolls were amazing. If you take up the invitation then we have succeeded in forming a collective belief. We will both then be disposed to make what Gilbert calls “collective belief statements” – for example by declaring this collective belief to some third party (“We think those spring rolls were amazing”).

Gilbert accounts for this sort of phenomenon by invoking the idea of a joint commitment. A joint commitment is a commitment of and by several people to their “acting as a body” in some way (where “acting” is to be understood broadly enough to include the having of an attitude).^13^ In the case of a collective belief that p, the parties jointly commit to believing that p as a body, or as Gilbert also puts it, to “emulate, as far as possible, a single [believer of p]” (2002a, 45). According to Gilbert, this form of commitment has important normative implications: the parties to any joint commitment owe it to one another to play their respective parts in seeing to it that they jointly fulfill the commitment. So if we jointly commit to believing, as a single body, that the spring rolls are delicious, then according to Gilbert each of us owes it to other to speak and act in accordance with our believing that, and each of us has the standing to rebuke the other for failing to fulfill that obligation. So if you suddenly start disparaging the spring rolls to a third party, I might pull you aside and rebuke you, saying “Hang on, didn’t we think they were amazing?”^14^

One important feature of Gilbert’s account of collective belief concerns the relation between collective belief and the personal beliefs of the parties involved. Gilbert is emphatic that her account differs from what she calls “summativism” about collective belief – the view that collective belief is just a matter of what all or most of the people included in the collective personally believe. By contrast, on Gilbert’s view it is neither necessary nor sufficient for the several parties to a collective belief to hold that same belief personally. For it may be that, in the situation described above, neither of us really likes the spring rolls at all, yet in virtue of our being jointly committed, we each incur the obligation to speak and act in line with our collectively believing the spring rolls were amazing. Conversely, both of us might personally believe the spring rolls were amazing, without this being something we are bound together in doing – in the sense that we owe each other our compliance.

Nevertheless, despite her emphatic non-summativism about collective belief, Gilbert is also clear that collective beliefs tend to have a profound influence over the personal beliefs of group members. According to her, collective beliefs have what Durkheim called “coercive power”, in the sense that group members will be strongly motivated to adopt the group view themselves. Gilbert explains this with reference to the way in which the parties to a collective belief make themselves liable to be rebuked by other parties if they do not toe the party line (as it were). She illustrates this with an example:

Bob and Judy collectively believe that the conservation of species is an important goal. When this collective belief was formed Judy herself personally believed the opposite. Now that the collective belief is in place, Judy recognises that Bob has the standing to rebuke her for expressing a view in conflict with it. This leads her to suppress any inclinations bluntly to assert that the conservation of species is unnecessary. Such inclinations initially come to her at times, but their incidence diminishes as a result of such regular suppression. Indeed, the belief that prompts them subsides. This will leave the field open for what she and Bob collectively believe to become her personal belief. If she regularly mouths the collective belief, or hears Bob mouthing it, this may well happen (Gilbert 2004, 99).

This example describes a familiar process. People do often join with others in letting some view stand as their collective view, despite their own personal disagreement or reservations. In doing so they may be motivated by sound social or political reasons, and they may privately resolve to maintain their personal (dissenting) view. However, once the collective belief is established, the pressures associated with being a party to that collective view can easily erode their personally held beliefs, so that they end up, *for no good epistemic reason*, sharing the view of the group. This seems to be what happens to Judy.

In the case of Judy and Bob the coercive power of collective belief is not deliberately exploited by Bob, nor is Judy cognizant of its impact on her. It just sort of *happens*. But it seem that the coercive power of collective belief could, in principle, be purposefully exploited by someone in order to influence either their own, or someone else’s, belief. In fact, Gilbert makes this point herself when she writes:

Getting others to join you in jointly accepting a certain view is a good way of making that view the personal view of those others […] and indeed, of oneself (Gilbert 1987, 198).

Since, according to Gilbert, the coercive power of collective belief can be harnessed, it seems plausible it could be harnessed as a tool for doxastic self-control.^15^ In other words, it seems as though someone could enter a joint commitment to believe something along with others precisely in order to coerce herself – in other words, to bring her future self – into maintaining that belief personally.

By way of example, imagine an amateur paleontologist living under an oppressive, staunchly creationist political regime. Based on her own testing and analysis of the fossils she has found, she discovers that, contrary to her society’s popular belief, the earth must be billions (rather than thousands) of years old. However, when she expresses this belief to others, most people either ridicule her, or threaten to report her to the authorities (there are severe penalties for such heresy). She does hear, from time to time, of similarly-minded people, but these are few and far between, and to her disappointment she finds that over time they too come to embrace the orthodoxy. She realizes that, in her current social and political climate, this may well happen to her, too: her rationally formed belief will be hard to maintain in the long run.

If our amateur paleontologist wishes to avoid that fate, one strategy open to her would be to join with other dissenters in establishing what they each hold personally – that the earth is billions of years old – as a collective belief. In this way they might each hope to harness the “co-ercive power” of collective belief as a tool for exercising diachronic doxastic self-control. This could be done informally, simply by chatting with like-minded people and mutually expressing readiness to let the view that the earth is billions of years old stand as their group view. Or it might be done more formally, by establishing some kind of “old-earth society”, dedicated to the promotion and defence of that particular view. Either way, the strategy is the same. In jointly committing to believing as a single body each party makes herself accountable to all the others for speaking and acting in accordance with the group view. The hope, then, is that the threat of one another’s criticisms or rebukes for non-compliance will be enough to counteract the political and social pressures to accede to the orthodox view. In this way, each party might succeed in sticking to their original, rationally formed belief.

## 5. The wrong kind of reasons?

So far I have described two forms of interpersonal epistemic commitment – Pettitian “avowal” and Gilbertian “collective belief” – that seem like they might be apt, in principle, be used as tools for diachronic doxastic self-control. To end off I want to consider the further normative question of whether using them for that purpose could be epistemically rational. Obviously, the answer one gives to this question depends on one’s conception of epistemic rationality. I will suggest that according to a certain strict form of evidentialism about epistemic rationality, these social mechanisms of doxastic self-control could not be considered epistemically rational, because they do not supply the right kind of reasons for belief. Yet from a slightly more relaxed perspective, these mechanisms may well be considered epistemically rational, since they serve epistemic goals – specifically, they promote true and responsible believing.

The strict version of evidentialism is widely held, but I think it is particularly well exemplified in the work of Pamela Hieronymi.^16^ According to Hieronymi, belief is a “commitment-constituted attitude”, in the sense that believing that p involves having settled for oneself (or being committed to an answer to) the question of whether or not p. This construal of belief gives a clear answer to the question what the “right kind” and “wrong kind” of reasons for believing are. The right kind of reasons are just those reasons that bear on the question that, when settled, amounts to the formation of a belief – that is, the question of whether or not some proposition is true. This is why only evidential reasons in support of p are proper epistemic reasons for believing that p: they are those reasons that bear positively on the question of whether p (is true). Any reasons that do not bear on that question are the wrong kind of reasons for believing. So, on this strict conception, believing that p because of the health or financial benefits of doing so is believing for the wrong kind of reasons, because the health or financial benefits of believing p do not bear on the question of whether or not p is true.

It seems to me that the social mechanisms of doxastic self-control considered here would fall afoul of this strict evidentialist conception of epistemic rationality. This is because of the *object* of the commitments involved in Pettitian avowal and Gilbertian collective belief – what those commitments are commitments *to*. Consider avowal first. On Pettit’s view, when you avow a belief, you “bet on yourself” to have and, within reason, to sustain the belief you avow. The object of this kind of commitment is therefore *attitude-centered* rather than *content-centered*: it is a commitment to the having of the belief, rather than a commitment to the truth of the content of that belief. Hence, what this attitude-centered commitment makes you accountable to others *for* is upholding the belief, for speaking and acting in line with it, or “living up to it”, as Pettit puts it.

But if this is the nature of the accountability incurred through avowal, then it doesn’t seem like the reasons that accountability can generate for sticking to your belief would, strictly speaking, be the right kind of reasons for belief. Instead, what undertaking that accountability provides one with is a certain kind of social incentive for maintaining the belief avowed. Once one has committed in the manner of avowal, one knows that there will be certain social costs associated with wavering or backtracking: these may be reputational losses, fewer opportunities for co-operation, and so on. But these social costs are not considerations that bear on the question of whether or not the content of the belief is true, and so cannot be counted as the right kind of reasons for believing, by the lights of the strict evidentialist.

Something similar appears to apply to Gilbertian collective belief. According to Gilbert, a set of people collectively believe that p if and only if they are jointly committed to believing that p as a single body, or to emulating, as far as possible, a single believer of p. Again, the object of the relevant commitment here is *attitude-centered* rather than *content-centered*. That is, the parties to this joint commitment are committed to upholding the belief in question, rather than being committed to the truth of the content of that belief. This shows up in the sort of accountability that the parties to a collective belief incur in relation to one another. They owe it to one another to speak and act in compliance with their collective belief, and they each have the standing to rebuke one another for failure to comply. This normative situation can then coerce or encourage the parties to a collective belief into maintaining that belief personally.

Here too it appears the interpersonal accountability incurred through collective belief does not generate what a strict evidentialist would consider the right kind of reasons for believing. It may well be that people are motivated to bring their personal beliefs into line with the beliefs of their groups. But the fact that your group has a certain belief – that you and others have seen fit to let some proposition stand as the view of the group – is not something that itself bears on the truth of the content of that belief. So while there may be certain social or psychological advantages to bringing one’s personal beliefs in line with the collectively held belief, these advantages are not, on the strict evidentialist view, the right kind of reasons for believing.

Considerations such as these make it tempting to dismiss the social mechanisms of doxastic self-management considered here as epistemically irrational. But I think that response would be too hasty. This is because although the reasons for sticking to one’s beliefs these mechanisms produce are not themselves the right kind of reasons for believing (by the lights of the strict evidentalist), they are also not entirely *non-epistemic* reasons either, on a par with the proverbial “health or financial” reasons for believing sometimes associated with non-evidentialism. More specifically, it seems that the interpersonal accountability a person incurs through avowal and collective belief is in an important sense an *epistemic* form of responsibility, and so may yet promote epistemically responsible, truth-conducive believing. If the fundamental goal of epistemic agency is the Jamesian one of forming and maintaining true rather than false beliefs, then there are reasons to think that avowal and collective belief can serve this goal, and to that extent can be considered epistemically rational.^17^

To see this, consider again the avowal of belief. According to Pettit, the avowal of belief makes a speaker responsible to others for “living up to [the belief avowed]”. But what exactly does it take to “live up to” a belief? As we have seen, Pettit himself stresses the need to speak and act in line with the belief, on pain of suffering the social costs associated with wavering or backtracking. But it could be argued that this represents only part of the picture – i.e. that truly “living up to” one’s belief is not only a matter of *maintaining the belief* but also a matter of fulfilling certain distinctively epistemic responsibilities associated with the belief avowed.^18^ When one avows a belief one not only projects a certain picture of oneself as a holder of that attitude, one also makes oneself liable to epistemic challenges (“Not p!”) or queries (“Why do you think that p?”) from one’s audience. In response to such challenges and queries, someone who has avowed their belief is under a certain amount of normative pressure to *answer* those challenges and queries, where this means providing properly epistemic reasons in reply – reasons that *do* bear on the question of whether the avowed belief is true. And here it is worth noting that, just as there are social costs associated with failing to maintain one’s avowed belief, so too there are social costs associated with *maintaining* one’s avowed beliefs in the face of unanswered challenges or criticism. So someone who suspends her belief in response to a justified challenge or legitimate query does not necessarily suffer a blow to her reputation; on the contrary, being open and responsive to others’ rational critique can actually enhance one’s reputation as a responsible epistemic agent.^19^

It is this dimension of *epistemic answerability*, and the way it encourages the maintenance of *defensible* belief, that I think may help to vindicate the rationality of belief-avowal as a strategy of doxastic self-control. When you avow a belief you make yourself answerable to others, in the sense that you knowingly undertake to offer reasons – proper epistemic reasons – in response to the challenges and queries of others. Though the prospect of such challenges and queries is not itself something that bears on the content of the beliefs you avow, it nonetheless provides a strong incentive to be apprised of those reasons. The upshot is that avowing one’s belief does indeed give one reason to maintain and defend the belief – not *come what may*, but only as long as the belief proves to be defensible. If we set strict evidentialism aside, it seems natural to count this as an epistemically rational form of doxastic self-control: in the terms adopted earlier, it is a matter of *staying true* rather than *holding fast*.

A similar point can be made with respect to collective belief. According to Gilbert, the parties to a collective belief that p owe it to one another to speak and act in line with that belief, or to “emulate, as far as possible, a single believer of p”. Like Pettit, Gilbert cashes this out in terms of *belief maintenance*. She claims that the primary obligations of the parties to a collective belief are that they play their respective parts in *upholding* that belief, by, for instance, not openly expressing disagreement, or by “act[ing] as would any of several *mouthpieces* of the body in question” (Gilbert 2002a, 45, emphasis in original). However, it is not clear that this is all there is, or should be, to “emulating a single believer of p”. After all, a single believer of p would not simply be required to voice the belief that p; she would also be epistemically answerable for that belief, in just the sense described above, of being liable to epistemic challenges and queries, and required to answer such challenges and queries with properly epistemic reasons.^20^

If this is right, then the establishment of collective belief, no less than the avowal of individual belief, involves the undertaking of a kind of epistemic answerability – here a kind of joint epistemic answerability, undertaken by and dischargeable by the group as a whole (the “plural subject of belief”, as Gilbert would call it). I think this is significant when considering the rationality of using collective belief as a tool for doxastic self-control. It is not obviously epistemically irresponsible to join with others in believing something, when one anticipates epistemic temptations that one would not be able to withstand alone. This need not be a matter of taking refuge within a community of like-minded people, in order to protect one’s belief. Instead, it can be a matter of taking an epistemic stand along with others, and facing the challenges to that belief together. As was the case with avowal, here too we find that the social reasons for sticking to one’s belief provided by being a party to a collective belief – reasons of solidarity or conformity – are not what a strict evidentialist would consider the right kind of reasons for believing. Yet because the group itself is epistemically answerable for its belief, these social reasons are still closely tied to the right kind of reasons for believing, and so can promote responsible, true believing. The parties to a collective belief have the standing to keep each other in line with respect to upholding the collective belief – not come what may, but only so long as they, together, can defend that belief on the basis of proper epistemic reasons. Insofar as this joint epistemic undertaking has a stabilizing effect on the members’ beliefs, this mechanism need not be dismissed as irrational. Instead, it can be seen as a matter of *staying true together*.

## 6. Conclusion

My aim in this paper was to explore the feasibility and rationality of using interpersonal epistemic commitments as mechanisms of doxastic self-control. I looked in particular at Philip Pettit’s account of belief-avowal, and Margaret Gilbert’s account of collective belief. With respect to the feasibility question, I followed Pettit and Gilbert in suggesting that these forms of interpersonal epistemic commitment make people responsible to one another for maintaining or living up to belief in question, and hence that they are apt tools of doxastic self-control. With respect to the rationality question, I explored something that is neglected by both Pettit and Gilbert, namely the way in which both belief-avowal and collective belief involve undertaking a distinctively epistemic form of answerability for the belief in question. It is this, I suggested, that may ultimately vindicate the rationality of these forms of commitment as tools for doxastic self-control.

If correct, I think this conclusion puts some pressure on the still widespread tendency to think of responsible epistemic agency as a fundamentally individual achievement. Our epistemic agency is not an isolated individual enterprise; it is socially situated and socially enacted. We rely on one another in myriad ways in our epistemic lives – to share our knowledge with one another; to model epistemic virtue; and, as I have suggested here, to hold one another epistemically responsible, and thereby help each other in staying true.

## Notes

1 See Paul (2015a, 2015b).

2 See note 11 below for references.

3 See, e.g., Heil (1984) and Mele (1987).

4 Classic arguments can be found in Williams (1973), who claims believing at will is conceptually impossible, and Alston (1988), who claims it is psychologically impossible.

5 These are the labels Peels (2017, Ch. 2) uses in his illuminating discussion of Alston’s distinction.

6 See Ginet (2001), Steup (2012) and Peels (2015) for three recent exceptions.

7 See e.g., Heil (1992), Feldman (2000), Nottelmann (2007). Interestingly, Peels (2017, 69) suggests indirect control is more frequently exercised for *belief maintenance* than belief formation.

8 Peels (2017) has argued that it is in virtue of our doxastic influence that we are fit to be held responsible for our beliefs. A number of others have made similar arguments using some notion of “control” that would, in terms of Alston’s distinction, more properly be called doxastic influence – see, e.g., Leon (2002), Pamela Hieronymi’s notion of “evaluative control” (2006, 2014) and Miriam Schleifer McCormick’s notion of doxastic “guidance control” (2015).

9 In a similar vein, Bernard Williams argues that we “construct our beliefs” and “steady our minds” because of the “presence and needs of other people”: [When] we are asked what our belief is, we may simply come out with an expression of one that is sincere in the most basic sense of its being spontaneous and uninhibited, and that fact itself will encourage us to stand by it, to present ourselves and go on presenting ourselves as people who have that view. (Williams 2002, 193)

10 Note that reporting and avowing do not exclude one another. On the contrary, when one reports one typically also avows, except that the content of one’s report and one’s avowal differ. For example, when one reports on some state of the world (say, that it is raining), then one typically avows the belief that the world is in that state (the belief that it is raining). In just the same way, when one reports that one believes that p, one (typically) thereby avows the second-order belief that one believes that p.

11 It is worth noting that Pettit’s idea, that avowal serves to stabilize attitudes, is at least in part an empirical claim. Are there empirical studies that support it? Pettit himself does not explicitly refer to any such data, but I think there is in fact a good deal of empirical literature that is in line with his proposal. Some of this literature concerns the way people shape or control their own minds by ascribing attitudes to themselves: put simply, people tend to form, maintain or go along with the attitudes that they have ascribed to themselves, even in cases where they did not, prior to the ascription, have the attitude ascribed. This is supported by so-called “confabulation” cases, in which subjects are led to falsely self-ascribe a preference, intention or desire, and then proceed to honour the self-ascribed attitude in their ongoing thought and behavior (see e.g., Gazzaniga 1985). According to Carruthers (2009, 127), “attributions of mental states, even if initially confabulated, are likely to be self-fulfilling. This is because agents will feel obliged to act in ways that are consistent with the mental states that they have attributed to themselves.” Also relevant to Pettit’s claim are the numerous social psychological studies that investigate how making various kinds of verbal commitment increases the likelihood of the speaker behaving and thinking in line with those commitments. For example, there are studies showing that people are more likely to follow through on medical treatment when they are made to promise they will do so (Kulik and Carlino 1987); there is research demonstrating the efficacy of pledges in fostering healthier food purchasing (Schwartz et al. 2014); there are studies that indicate that recycling practices and pro-recycling attitudes can be promoted by getting people to enter written agreements (Schultz, Oskamp, and Mainieri 1995); and there are studies that have shown the efficacy of oath-taking in inducing truthfulness (Jacquemet et al. 2013). The idea behind many of these studies is that people have a basic drive or bias towards consistency, and hence will be strongly motivated to think and act in line with their professed commitments. As Cialdini (2009, 52) puts it: “Once we have made a choice or taken a stand, we will encounter personal and interpersonal pressures to behave consistently with that commitment. Those pressures will cause us to respond in ways that justify our earlier decision.” (Thanks to a reviewer for encouraging me to address this point.)

12 Gilbert (1987, 1994, 2002a), Gilbert and Pilchman (2014).

13 Gilbert (2002b).

14 One might wonder what collective belief *really* amounts to, given that it does not require the parties to hold that belief themselves. I think Gilbert’s answer to this question would be that collective belief amounts to a complex normative situation – a package of rights and responsibilities on the part of the parties to a collective belief – that is brought into existence by the formation of a joint commitment. A number of philosophers have found this kind of response unsatisfying, however. More specifically, they do not see why collective belief should be called “belief” at all, since it does not aim at truth and can be formed voluntarily (see esp. Wray 2001; Meijers 2003). In recent work, Gilbert has conceded some of this criticism (Gilbert and Pilchman 2014) and accepted that collective belief may not share all the features of individual belief. I do not think this debate over whether collective belief is belief properly so-called affects my discussion here. The question is simply whether the phenomenon described and analysed by Gilbert (whether or not we wish to label it *collective belief*) can be mobilized by individuals as a tool for doxastic self-control.

15 Like Pettit, Gilbert does not cite empirical literature in support of her claim that collective belief tends to have a profound influence on individual belief. Although it is not exactly clear just what relation it bears to Gilbert’s notion of collective belief, some relevant empirical research may be found within the literature on “groupthink” (Janis 1982; Esser 1998). Groupthink concerns the way in which certain groups or organizations establish consensus by avoiding conflict and suppressing dissenting views. There is evidence that suggests that groupthink can lead not just to the behavioural compliance of members with the established group views but also to their “internalization” of these views. In other words, groupthink can lead members to bring their attitudes into line with the collectively-held attitudes (see McCauley 1989). According to Janis (1971, 85), “The more cohesive the group, the greater the inner compulsion on the part of each member to avoid creating disunity, which inclines him to believe in the soundness of whatever proposals are promoted by the leader or by a majority of the group’s members.”

16 See esp. Hieronymi (2005). It should be pointed out that, in contrast to some *even stricter* evidentialists, Hieronymi does accept that the wrong kind of reasons for belief are still genuine reasons for belief (see Reisner 2009, who for this reasons dubs Hieronymi an “ecumenical evidentialist”).

17 I am grateful to a reviewer for suggesting this line of reasoning.

18 In (Townsend forthcoming) I criticize Pettit for neglecting this aspect of epistemic answerability.

19 Thanks to a reviewer for suggesting this point.

20 In previous work (Townsend 2016) I have criticized Gilbert’s account of collective belief for its failure to accommodate this epistemic kind of answerability.
